# Dissecting Agronomically favorable genotypes in temperate *japonica* rice via haplotype analysis of a Japan‐MAGIC population

**DOI:** 10.1111/tpj.70936

**Published:** 2026-06-04

**Authors:** Hirofumi Fukuda, Akari Fukuda, Toshihiro Sakamoto, Yoshihiro Kawahara, Ken Naito, Taiji Kawakatsu, Jun‐ichi Yonemaru, Daisuke Ogawa

**Affiliations:** ^1^ Research Center for Agricultural Information Technology National Agricultural and Food Research Organization (NARO) Tsukuba Ibaraki 305‐0856 Japan; ^2^ Institute of Crop Science NARO Tsukuba Ibaraki 305‐8518 Japan; ^3^ Institute for Agro‐Environmental Sciences NARO Tsukuba Ibaraki 305‐8604 Japan; ^4^ Research Center for Advanced Analysis NARO Tsukuba Ibaraki 305‐8602 Japan; ^5^ Research Center of Genetic Resources NARO Tsukuba Ibaraki 305‐8602 Japan; ^6^ Institute of Agrobiological Science NARO Tsukuba Ibaraki 305‐8604 Japan; ^7^ BioResource Research Center RIKEN Tsukuba Ibaraki 305‐0074 Japan

**Keywords:** *Oryza sativa*, elite cultivar, quantitative trait locus (QTL), combinational genotypes, breeding history

## Abstract

Crop breeding assembles genomic variants into cultivars via crossing and selection. Phenotypic selection has improved yield and lodging tolerance but has limited genetic insights. We show how specific genomic variants and their combinations underpin advances in modern rice breeding in Japan. Through genome‐wide association study using a multi‐parent advanced‐generation intercross population derived from four temperate *japonica* cultivars, we identified 11 quantitative trait loci (QTLs) for key agronomic traits, including days to heading, shoot biomass, panicle length, and culm length under field conditions. *GA20ox1*, *GA20ox2*, and *Hd1* were among the QTLs, and their natural variants were well conserved in temperate *japonica* cultivars bred in Japan, underscoring distinct selection pressures at these loci. By integrating genotype data with 5‐year yield performance evaluation trials of elite cultivars, we found that cultivars carrying multiple‐copy *GA20ox1* and functional *Hd1*, together with non‐functional *ga20ox2*, tended to have shorter culms and higher grain yield than cultivars with multiple‐copy *GA20ox1*, functional *Hd1*, and *GA20ox2*. This yield advantage was consistent across latitudes in Japan. These results reveal favorable genotype combinations underlying modern *japonica* improvement and provide a genomic framework for breeding semi‐dwarf, high‐yielding cultivars adapted to temperate rice‐growing regions in Asia.

## Significance Statement

How modern temperate *japonica* rice achieved higher yield with stronger lodging resistance remains unclear at the level of interacting genetic variants. Using a Japan‐derived multi‐parent intercross population and 5‐year trials of elite cultivars, we identify favorable *GA20ox1*–*GA20ox2*–*Hd1* genotype combinations that shorten culms yet increase yield across Japanese regions, providing practical targets for breeding climate‐resilient, semi‐dwarf rice for temperate Asia.

## INTRODUCTION

Rice (*Oryza sativa* L.) is a staple food for more than half of the global population, particularly in temperate regions, including Japan, making its genetic improvement a global priority for ensuring food security under increasing environmental and demographic pressures. To meet rising food demand, breeding programs have developed cultivars that not only have high yield potential but are also resistant to lodging owing to their semi‐dwarf phenotypes (Hargrove et al., 1988; Hargrove & Cabanilla, 1979). Landmark cultivars such as IR8, developed by International Rice Research Institute in the 1960s, and the Japanese cultivar Reimei, derived from gamma‐ray‐induced mutants of Fujiminori, played pivotal roles in enhancing lodging resistance through semi‐dwarf phenotypes and served as key parental lines in subsequent Japanese breeding efforts (Ashikari et al., 2002; Futsuhara et al., 1967). The semi‐dwarf characteristics of these cultivars were later attributed to distinct mutations in *Sd1*/*GA20ox2*, which encodes a key enzyme in the late stage of gibberellin biosynthesis (Ashikari et al., 2002; Spielmeyer et al., 2002). However, rice breeding in Japan from the 1960s to early 2000s relied heavily on empirical phenotypic selection, limiting insights into the genetic basis of agronomic traits. To advance breeding strategies and improve elite cultivars, it is essential to identify the genetic variants that govern key agronomic traits, including semi‐dwarf architecture.

Dwarfism in rice can depress canopy photosynthesis and decrease biomass, because total leaf area is reduced and leaves are packed more densely, thus increasing self‐shading and lowering light‐use efficiency (reviewed by Weng et al., 2025). As canopy photosynthesis reflects the integrated performance of all leaves, leaf size and spatial arrangement determine how much leaf area can be deployed and how effectively light is intercepted. Semi‐dwarf plants carrying the *ga20ox2* allele typically have shorter leaves and reduced stature (Feng et al., 2023; Sasaki et al., 2002), and this can constrain photosynthetic capacity in dense canopies. Breeding practice therefore likely favored *ga20ox2*‐possessing lines that maintain leaf length and canopy height (CH) after crossing with core semi‐dwarf cultivars. In Japan, culm length (CL) has remained broadly constant for decades, whereas biomass and grain yield have increased (Matsushita et al., 2024). This trend indicates that breeders consequently paired *ga20ox2* alleles with genotypes that sustain shoot growth on the basis of phenotypic selection, thus achieving higher biomass and yield without sacrificing lodging resistance.

Genome‐wide association studies (GWAS) using rice populations have been instrumental in identifying quantitative trait loci (QTLs) associated with flowering time, plant architecture, yield‐related traits, and responses to abiotic and biotic stresses (Serrie et al., 2025; Sertse et al., 2021; Yano et al., 2016). These studies highlight the power of high‐resolution mapping to uncover natural variations relevant to breeding. However, the use of highly structured natural accessions can introduce confounding due to population stratification (Cardon & Palmer, 2003; Lander & Schork, 1994) and cryptic relatedness (Sillanpää, 2011), potentially leading to spurious associations. To address these limitations, multi‐parental populations such as multi‐parent advanced‐generation intercrosses (MAGIC) have emerged as powerful resources for the genetic dissection of complex traits (McMullen et al., 2009; reviewed by Huang et al., 2015; Abdelraheem et al., 2021). MAGIC populations are generated through controlled intercrossing among multiple founder lines, thus helping to minimize hidden population structure and maximize recombination. In this way, they combine broad genetic diversity with high mapping resolution and reduced confounding effects. Previously, we conducted haplotype‐based GWAS by using a mixed *indica*–*japonica* MAGIC population consisting of eight founder lines derived from Japanese cultivars (i.e., Japan‐MAGIC1, JAM1), and we successfully identified well‐known genes and novel QTLs associated with glutinous endosperm, plant spreading habit, CL, aboveground dry weight (AGW, i.e., shoot biomass), panicle weight (PW), and grain shape (Ogawa et al., 2021; Ogawa, Nonoue, et al., 2018; Ogawa, Yamamoto, et al., 2018). We recently developed a new MAGIC population, Japan‐MAGIC2 (JAM2), derived from four elite temperate *japonica* founders bred in Japan in the 21st century (Taniguchi, Sakamoto, et al., 2025). Two‐year field evaluations demonstrated that the JAM2 population consistently had broad phenotypic diversity, indicating that it harbors a wide range of natural variants underlying modern elite temperate *japonica* cultivars.

In this study, we aimed to identify which natural variants have been used throughout the Japanese breeding history of temperate *japonica* cultivars and to elucidate their roles in the development of modern Japanese cultivars. To explore the natural variants, we first conducted haplotype‐based GWAS by using the JAM2 population and identified haplotypes/QTLs associated with agronomic traits important for lodging resistance and yield. We then conducted data mining of DNA sequencing results, including whole‐genome sequencing, to identify the natural variations in candidate genes underlying the identified QTLs. Finally, by using Japanese historical field records of elite temperate *japonica* cultivars, we validated the roles of combinational genotypes of these candidates in agronomic traits important for lodging resistance and yield.

## RESULTS

### Genomic information on founders of the Japan‐MAGIC2 (JAM2) population and JAM2 lines

**Figure 1:**
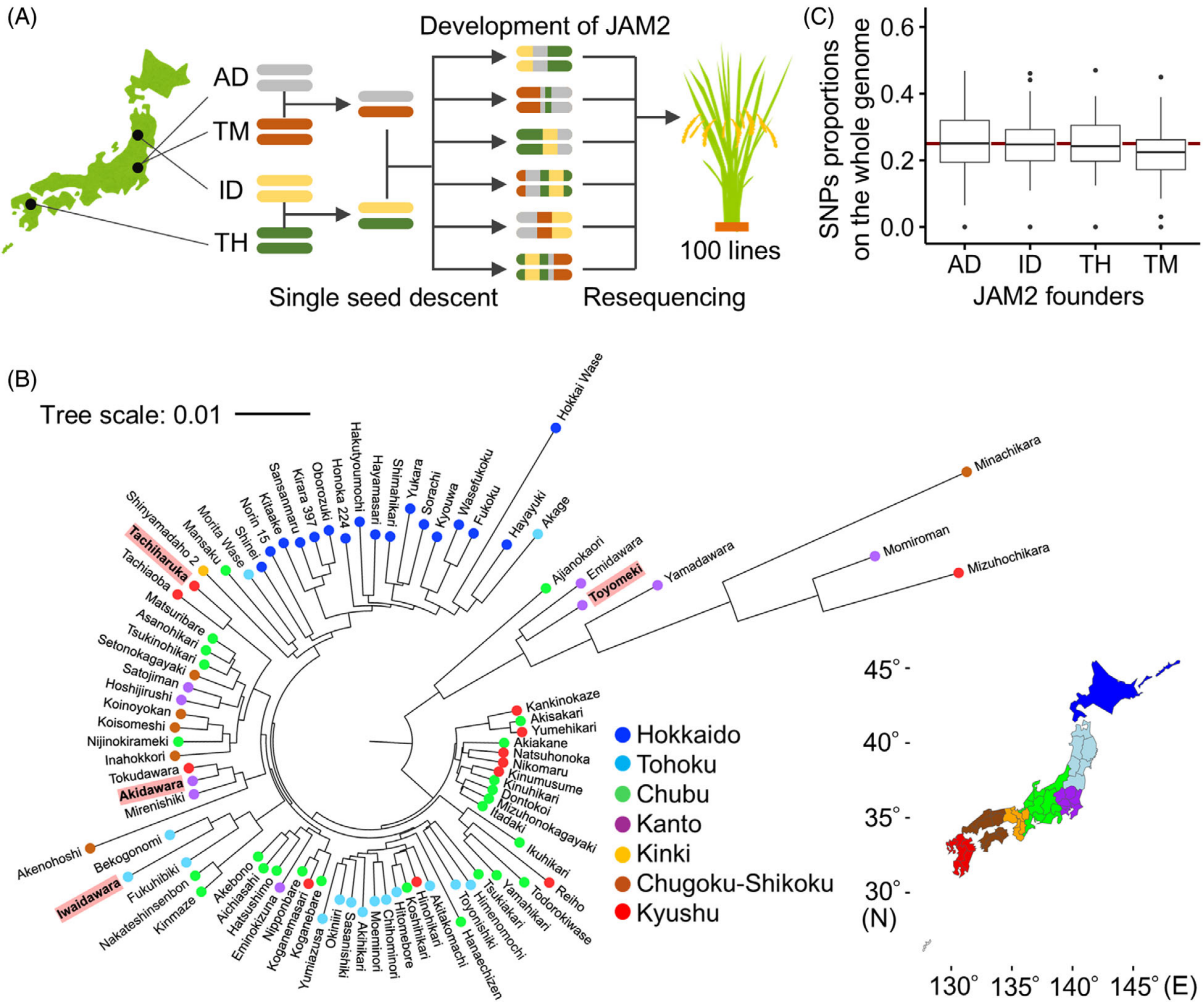
Genomic information on JAPAN‐MAGIC2 founders and the JAPAN‐MAGIC2 population. (A) Schematic overview of the process used to establish the JAM2 population, consisting of 100 lines derived from four temperate *japonica* cultivars: Akidawara (AD), Iwaidawara (ID), Tachiharuka (TH), and Toyomeki (TM). (B) Phylogenetic tree of temperate *japonica* rice cultivars bred in Japan. Colored circles indicate the regions where each cultivar was developed. The four founder cultivars of the JAM2 population are highlighted. (C) Proportions of JAM2‐founder‐derived single nucleotide polymorphisms (SNPs) in the whole genome of JAM2 lines; 131 537 SNPs were used for this analysis.

The JAM2 founders (i.e., AD, ID, TH, and TM) originated from distinct pedigrees and were bred in regions from northeastern to southwestern Japan (Figure 1A,B; Figure S1). We analyzed the whole‐genome resequencing data of a total of 100 JAM2 lines (F5 in 2022, F6 in 2023) and performed short‐read genome assemblies to detect SNPs. We found 131 537 SNPs in all the chromosomes (Table S1). The proportions of SNPs derived from each founder were, in descending order, AD, ID, TH, and TM, with an almost equal distribution of approximately 25% from each founder on the whole genome (Figure 1C). The distribution of SNPs differed among the founders at an individual chromosome level (Figure S3). This indicated that various genomic variants were introgressed among the JAM2 lines.

### QTLs associated with agronomic traits in the JAM2 population and their candidate genes

**Figure 2:**
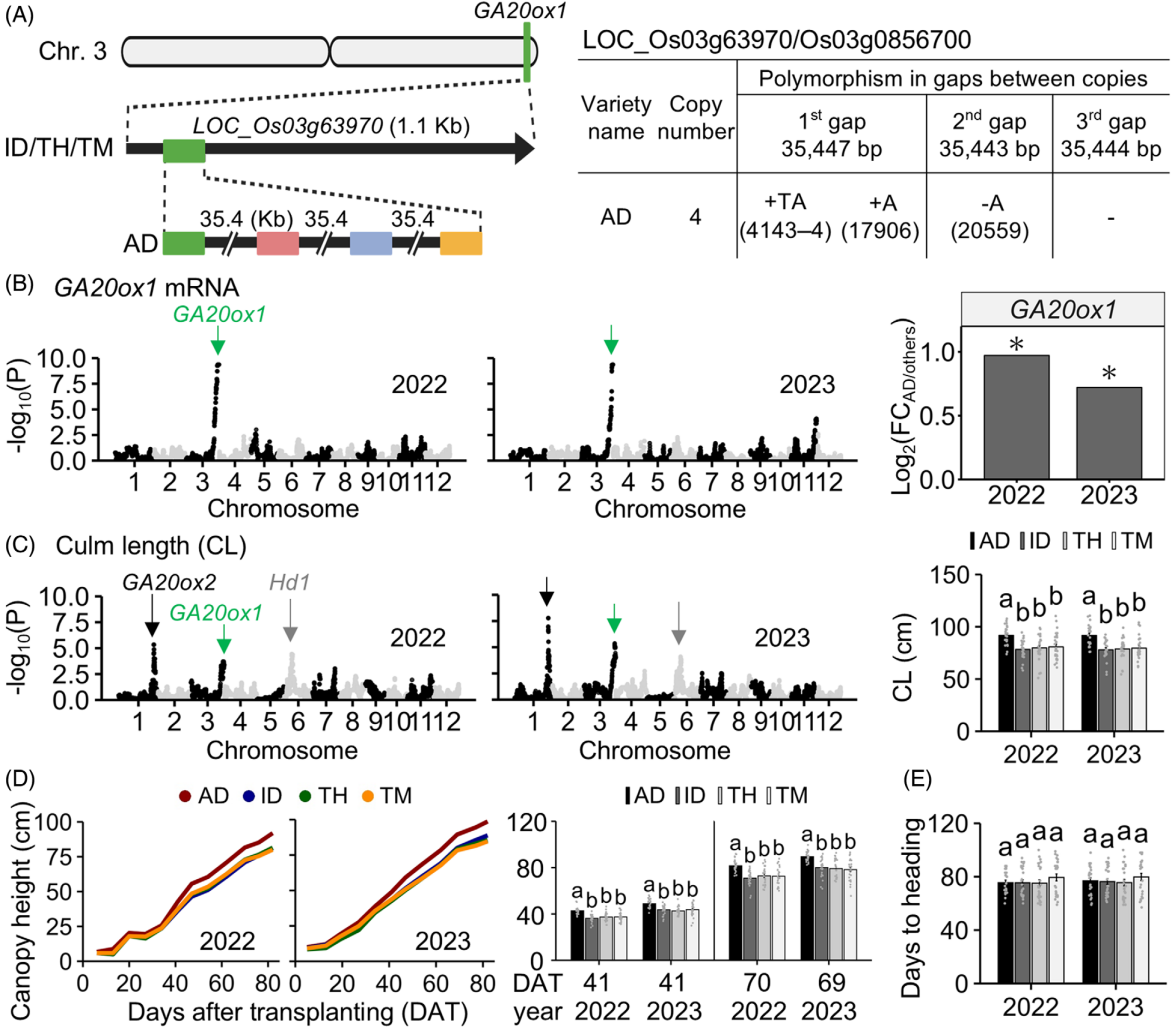
Effect of a putative QTL harboring a new genotype of *GA20ox1* on culm length and canopy height at the vegetative stage. (A) Scheme of the *GA20ox1* (LOC_Os03g63970/Os03g0856700) genotype among the four founders (AD, Akidawara; ID, Iwaidawara; TH, Tachiharuka; TM, Toyomeki). Polymorphisms in three gaps of the *GA20ox1* tandem repeats are described. Numbers in parentheses indicate positions counted from the first nucleotide (+1) in each gap. (B) Expression genome‐wide association study (GWAS) of *GA20ox1* among the JAM2 lines. Differences in the expression levels of *GA20ox1* between single and four copies in the JAM2 lines are shown. Asterisks indicate significant differences between genotypes of multiple copies derived from AD and a single copy derived from the other three founders (*P*adj < 0.05, *n* = 23–77). (C) GWAS detected a peak including the *GA20ox1* locus for culm length (CL) among the JAM2 lines. (D) Time series of canopy height (CH) at the vegetative stage and (E) days to heading (DTH) in JAM2 lines classified by the haplotype including the *GA20ox1* locus. Plants were transplanted to experimental fields on May 18, 2022 and May 17, 2023. Time series of CH are shown, with means of each group classified with *GA20ox1* genotypes derived from each JAM2 founder. The means ± SD for CL, CH at two time points, and DTH are shown (*n* = 23–77). Different letters indicate significant differences among JAM2 lines in a haplotype‐dependent manner (*P* < 0.05, Tukey–Kramer test). FC, fold change.

The JAM2 population showed variations in DTH, CL, PL, PN, PW, SLW, and AGW under experimental field conditions in 2022 and 2023 (Figure S4a), as partly described in our previous study (Taniguchi, Sakamoto, et al., 2025). Haplotype‐based GWAS revealed 11 QTLs [4 > −log_10_(*P*) in both 2022 and 2023] related to DTH, CL, PL, SLW, and AGW (Figure S4b,c, Table 1). Among the QTLs, some haplotypes included loci of agronomically important genes: *Grain number 1a* (*Gn1a*, LOC_Os01g10110/Os01g0197700) (Gouda et al., 2020; Kim et al., 2016; Li et al., 2016) for PL; *Suppressor of overexpression of constans 1* (*SOC1*, LOC_Os03g03070/Os03g0122600) (Lee et al., 2004) for DTH, *Heading date 1* (*Hd1*, LOC_Os06g16370/Os06g0275000) (Yano et al., 2000) for DTH, CL, and AGW; and *Gibberellin 20‐oxidase 1* (*GA20ox1*, LOC_Os03g63970/Os03g0856700) and *Gibberellin 20‐oxidase 2* (*GA20ox2*, LOC_Os01g66100/Os01g0883800) for CL (Table 1). Among these five genes, we found novel natural variants of *Gn1a* (Figure S5), *SOC1* (Figure S6), and *GA20ox1* (Figure 2). We found that newly identified natural variants of *Gn1a* and *SOC1* were used rarely in the past in Japanese breeding of staple rice cultivars (See Supplementary documentation S2). Variants of *GA20ox1*, *GA20ox2*, and *Hd1* for CL were used frequently in modern temperate *japonica* cultivars (details described later).

**Table 1:** Identification of putative QTLs for agronomic traits in the JAM2 population

(QTL) No	Chr	Position	Trait	2022	2023	Candidates	Variant reference
I	1	4 958 120–4 979 170	PL	1.98E‐04	6.00E‐05	*Gn1a*	This study
II	1	38 398 436–38 513 991	CL	1.93E‐04	1.41E‐06	*GA20ox2*	Sasaki et al., 2002
III	3	225 264–2 403 133	DTH	1.47E‐04	1.02E‐04	*SOC1*	This study
IV	3	33 002 789–33 034 128	CL	3.45E‐04	3.86E‐05		
V	3	35 099 038–35 113 003	CL	1.17E‐04	2.21E‐06	*GA20ox1*	This study
VI	6	7 235 289–7 253 747	DTH	2.08E‐08	1.17E‐08		
VII	6	9 333 525–9 524 731	DTH	6.22E‐13	3.86E‐13	*Hd1*	Yano et al., 2000
VII	6	8 987 955–9 303 179	CL	7.78E‐05	4.16E‐06	*Hd1*	
VII	6	9 333 525–9 575 842	AGW	1.56E‐11	2.59E‐09	*Hd1*	
VII	6	9 315 860	SLW	5.44E‐12	6.19E‐12	*Hd1*	
VIII	6	21 439 006–21 452 322	SLW	1.11E‐04	4.35E‐05		
IX	6	23 003 215–23 056 380	DTH	1.65E‐05	1.30E‐05		
X	6	23 920 500	SLW	3.95E‐05	1.63E‐05		
XI	7	20 826 800–20 844 742	DTH	8.60E‐04	6.70E‐04		

Summary of putative QTLs detected in this study. Positions indicate the genomic region with *P‐*values < 0.001 [4 > −log_10_(*P*)] in both 2022 and 2023 among the JAM2 lines. Candidate genes were determined on the basis of their known roles in each agronomic trait in previous studies.

DTH, days to heading; CL, culm length; PL, panicle length; PN, panicle number; PW, panicle weight; SLW, stem and leaf dry weight; AGW, aboveground dry weight.

### Positive role of a natural variant of multiple‐copy *GA20ox1* in increasing CH under field conditions

Without SNPs or indels for *GA20ox1* among the JAM2 founders, we conducted long‐read sequencing by using high‐molecular‐weight DNA from each JAM2 founder. We found four tandem copies of *GA20ox1* in AD, whereas the others had only one copy (Figure 2A). GWAS analysis revealed a single prominent peak near *GA20ox1* (Figure 2B). When we grouped the JAM2 lines in a haplotype‐dependent manner, the mRNA levels of *GA20ox1* in the lines harboring the tandem repeat of *GA20ox1* (*GA20ox1* ^AD^) were significantly higher (*P*adj < 0.05) than those in the other lines with a single copy (*GA20ox1* ^ID/TH/TM^). These mRNA analyses indicated that the higher copy number of *GA20ox1* was responsible for the high gene expression level among the JAM2 lines. Our GWAS analysis revealed several prominent peaks near *GA20ox1* (Figure 2C). CL at harvest was significantly longer in *GA20ox1* ^*AD*^ than in *GA20ox1* ^ID/TH/TM^ among the JAM2 lines. Time‐series investigation of CH revealed that the *GA20ox1* ^*AD*^ variant significantly increased CH from 41 days after transplanting (DAT) in both 2022 and 2023 (Figure 2D); this was more than a month before heading, the time of which did not differ among the lines (Figure 2E). Differences in the *ga20ox2* variant had little effect on CH at 41 DAT in 2022, although the effect of the distinct genotypes was observed at about 70 DAT in both 2022 and 2023 and at 41 DAT in 2023 (Figure S7a,b). These results suggested that CH differences arose earlier with the multiple‐copy *GA20ox1* variant than with the non‐functional *ga20ox2* variant.

### Positive effect of *Hd1* on vegetative growth under field conditions

We found a 36‐bp insertion in the first exon in TM and a 36‐bp insertion with a 43‐bp deletion in the first exon in ID (Figure S8a). These variants had already been reported, and they cause early heading, indicating that the genotype is functionally weak (Yano et al., 2000). JAM2 lines harboring haplotypes including the loci of ID‐ and TM‐type *hd1* had significantly shorter DTH, lower AGW, and shorter CL than those of AD‐ and TH‐type *Hd1* (Figure S8b). Transcriptome analysis showed that the mRNA levels of *early heading date 1* (*Ehd1*, LOC_Os10g32600/Os10g0463400), *heading date 3a* (*Hd3a*, LOC_Os06g06320/Os06g0157700), and *RICE FLOWERING LOCUS T 1* (*RFT1*, LOC_Os06g06300/Os06g0157500) were significantly higher in lines harboring ID‐ and TM‐type *hd1* than in those with AD‐ and TH‐type *Hd1* (Figure S8c). Together, our results suggested that *Hd1* was a causal gene in the DTH/AGW/CL‐related QTLs on chromosome 6 among the JAM2 lines. To validate the exact timing of when the difference in *Hd1* alleles affects shoot biomass, we compared these traits between Koshihikari and a near‐isogenic line (NIL) harboring the Kasalath‐derived functionally weak *hd1* allele in the Koshihikari genetic background (Itoh et al., 2018). Plant height and AGW differences appeared during the period from the heading date of the NIL to that of Koshihikari (Figure S8d,e). The accumulating evidence indicated that shoot biomass production is enhanced through delayed heading timing controlled by the Hd1–Ehd1–Hd3a/RFT1 pathway.

### Well‐conserved genotypes of *GA20ox1* , *GA20ox2* , and *Hd1* in temperate *japonica* cultivars

**Figure 3:**
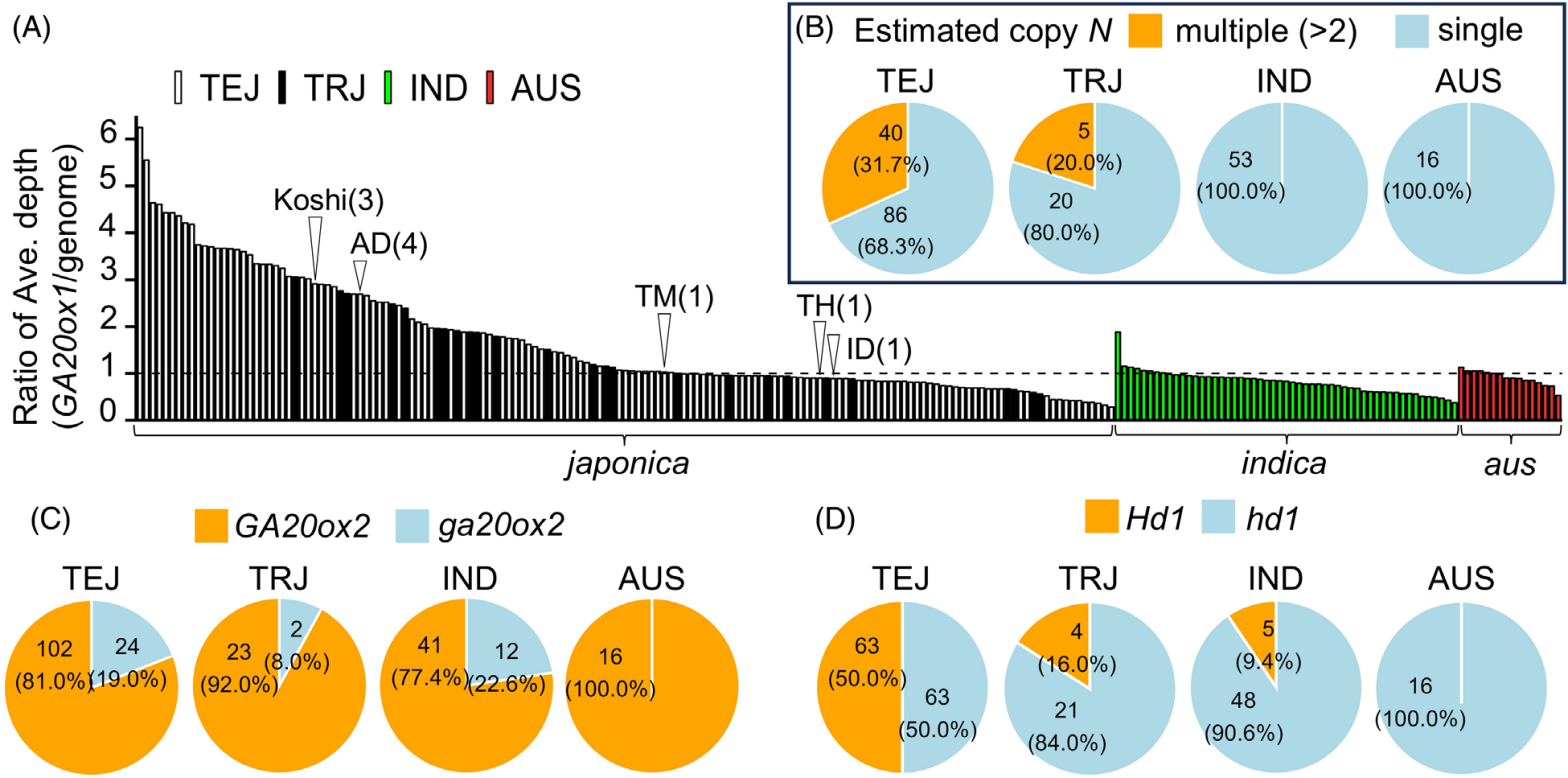
Conserved ratios of natural variations of multiple‐copy *GA20ox1*, the IR8‐derived deletion‐type *ga20ox2* allele, and the functionally weak allele *hd1* with a 36‐bp insertion in the first exon among different rice subspecies. (A) Ratio of the average depth of the *GA20ox1* locus to that of the whole genome. Numbers inside the circles represent the copy numbers identified for each JAM2 founder (in this study) and for Koshihikari (“Koshi”) in a previous study (Park et al., 2025). Proportions are shown of different genotypes of (B) *GA20ox1*, (C) *GA20ox2*, and (D) *Hd1*. Multiple‐copy *GA20ox1* genotypes were estimated with a threshold of more than a twofold change in the ratio of average depth (*GA20ox1* locus vs. the whole genome). *ga20ox2* was marked by a >380‐bp deletion in the first exon. *hd1* was marked by a 36‐bp insertion in the first exon. Short‐read resequencing data of 220 rice cultivars provided by the NARO (National Agriculture and Food Research Organization) Genebank (TASUKE+) were used in the analyses of the three genotypes. TEJ, temperate *japonica*; TRJ, tropical *japonica*; IND, *indica*; AUS, *aus*.

Next, we explored the degree of conservation of the natural variants we found in JAM2 (i.e., multiple‐copy *GA20ox1*, deletion‐type *ga20ox2* allele, insertion‐type *hd1* allele) among different rice subspecies. As the long‐read sequencing data were quite limited in rice, including Japanese cultivars, in the published dataset (Jain et al., 2019; Sugimura et al., 2024; Tanaka et al., 2020; Wang et al., 2023), we estimated whether the copy number variation of *GA20ox1* was single or multiple by using the ratio of the average depth in the *GA20ox1* locus to the whole genome, based on the short‐read resequencing data of 220 cultivars. Despite overestimation of the copy number differences, this ratio maximizes the sensitivity of finding multiple‐copy‐type natural variants. Homozygous multiple‐copy‐type cultivars such as Koshihikari (Park et al., 2025; Wang et al., 2023) and AD had more than twofold the average depth ratio of ID, TH, and TM, which have a homozygous single copy (Figure 3A); this was reasonable considering our long‐read sequencing results (Figure 2A). These tendencies indicated that our analysis captured the differences in the ratios of average depth in the *GA20ox1* locus to the whole genome due to the copy number variation in the JAM2 founders. With a threshold of 2, the proportion of putative multiple‐copy‐type cultivars inside the *japonica* population was 20% (5/25) in the tropical *japonica* subgroup and 32% (40/126) in the temperate *japonica* subgroups (Figure 3B; Table S3). With this threshold, putative multiple‐copy‐type cultivars were not included in the *indica* subgroup (*indica* and *aus*). These results suggested that multiple copies of *GA20ox1* are present mainly in the *japonica* subgroup, and especially in temperate *japonica* cultivars. The *ga20ox2* natural variant conserved in AD contains a 383‐bp deletion in the first exon (Figure S7a). This variant originated from the Taiwanese cultivar Dee‐geo‐woo‐gen and is conserved in IR8, a pedigree ancestor of AD (Chandler, 1968; Ferrero‐Serrano et al., 2019). We found that this deletion‐type variant was conserved in 38 of 220 cultivars, with the highest proportion in temperate *japonica* (24 cultivars) (Figure 3C; Table S4). For *Hd1*, functionally strong alleles without a 36‐bp insertion in the first exon were included in half (63/126) of temperate *japonica*, but they were much less common in tropical *japonica*, *indica*, and *aus* (9/94) (Figure 3D; Table S5). These patterns indicated that there was preferential retention of non‐functional *ga20ox2* and functional *Hd1* alleles in temperate *japonica* breeding compared with that in tropical regions.

### Historically selected *GA20ox1* and *GA20ox2* combinational genotypes underlying shorter CL and higher yield in elite Japanese rice cultivars

**Figure 4:**
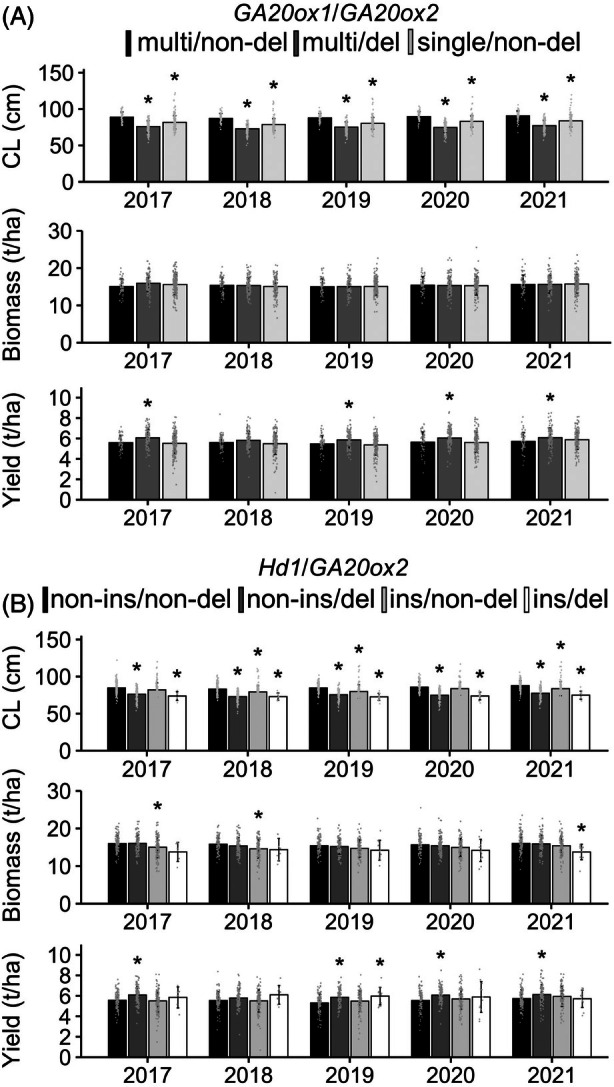
Effects of combinational genotypes of *GA20ox1*, *GA20ox2*, and *Hd1* on culm length, biomass, and yield in Japanese cultivars. Culm length (CL), shoot biomass, and grain yield among Japanese cultivars classified by combinational genotypes of (A) *GA20ox1* or (B) *Hd1* with *GA20ox2* under the experimental Japanese field conditions from 2017 to 2021 are shown. Each point represents the mean per cultivar × site × year for each agronomic trait, based on historical field records obtained by NARO. A total of 44 cultivars tested at up to total of 69 sites are plotted. Means ± SD are shown (CL and yield, *n* = (a) 53–187, (b) 6–124; biomass, *n* = (a) 50–179, (b) 6–121). Asterisks indicate significant differences from the control combinational genotypes: multiple‐copy *GA20ox1* or *Hd1* allele without a 36‐bp insertion in the first exon combined with the *GA20ox2* allele without a >380‐bp deletion in the first exon (i.e., combinations of each of the functionally strong genotypes) (*P* < 0.05, Dunnett's test). multi, multiple‐copy; non‐del, non‐deletion; del, deletion‐type mutation; non‐ins, non‐insertion; ins, insertion‐type mutation.

Our CH results via UAV‐based time‐series analyses suggested that the presence of multiple copies of *GA20ox1* has the potential to alleviate the *ga20ox2*‐dependent negative impacts on shoot growth at the vegetative stage (Figure 2D, E; Figure S7b,c). We then evaluated the combinational effects of the *GA20ox1* and *GA20ox2* genotypes on CL, shoot biomass, and yield by using the available historical field records collected between 2017 and 2021 for 48 Japanese cultivars. These cultivars' names were registered from 1936 to 2020. Historical field record analysis showed that CL was significantly shorter in the multiple‐copy *GA20ox1* and deletion‐type *ga20ox2* combinational genotypes (multi/del) and the single‐copy *GA20ox1* and *GA20ox2* combinational genotypes (single/non‐del) than in the multiple‐copy *GA20ox1* and *GA20ox2* combinational genotypes (multi/non‐del) from 2017 to 2021 (Figure 4A, upper), indicating that our dataset validated the known positive effects of *GA20ox1* and *GA20ox2* on CL (Ashikari et al., 2002; Oikawa et al., 2004; Sasaki et al., 2002). Shoot biomass was not changed by the variations in the combinational genotypes (Figure 4A, middle). Notably, yield was significantly higher in the multi/del genotypes than in the multi/non‐del ones in 4 of the 5 years, with a similar increasing trend observed in the remaining year (Figure 4A, lower). Such a decreasing trend in CL and an increasing trend in yield were observed from the Tohoku to the Kyushu regions of Japan (Figure S9a,b).

### Historically selected *Hd1* and *GA20ox2* combinational genotypes contributing to shorter CL and higher yield

As in the multiple‐copy *GA20ox1* natural variant, *Hd1* alleles without an insertion‐type mutation in the first exon enhanced shoot growth (Figure S8b,e). Thus, these functionally strong alleles may ameliorate the negative impact of *ga20ox2* on shoot biomass. Our analysis of historical field records revealed that, in at least 3 of the 5 years, when each of the genotypes was the insertion‐type mutation of *hd1* (ins) or the deletion‐type mutation of *ga20ox2* (del), CL was significantly shorter than that of the functionally strong combinational genotypes of *Hd1* and *GA20ox2* (non‐ins/non‐del) (Figure 4B, upper). Biomass had a decreasing tendency in cultivars harboring the *hd1* mutations (ins), and the combinational genotype of *hd1* and *ga20ox2* mutations (ins/del) compared with those harboring non‐ins/non‐del ones across the 5 years (Figure 4B, middle). Yield was significantly greater in the non‐ins/del genotypes than in the non‐ins/non‐del ones in 4 of the 5 years (Figure 4B, lower). The same trends of decreased CL and increased yield were observed from the Tohoku to the Kyushu regions of Japan as well as in the multi/del genotypes of *GA20ox1* and *GA20ox2* (Figure S9a,b).

### Historical and geographical distribution of combinational genotypes of *GA20ox1* , *GA20ox2* , and *Hd1* for high‐yield and shorter CL across Japanese rice‐growing regions in past breeding programs

**Figure 5:**
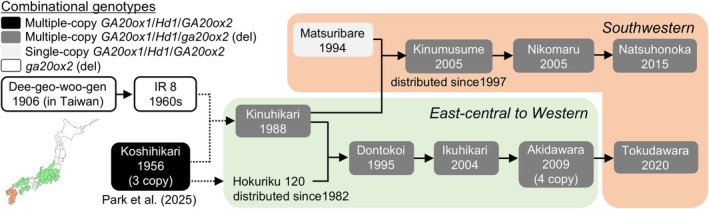
Historical emergence of combinational genotypes based on multiple‐copy *GA20ox1* and functionally strong *Hd1* genotypes with the IR8‐derived deletion‐type *ga20ox2* allele in Japanese breeding programs. The pedigrees of Japanese cultivars harboring different combinational genotypes of *GA20ox1*, *Hd1*, and *GA20ox2/Sd1* are shown. These cultivars were bred across east‐central to western regions (light green) or southwestern regions (light orange) of Japan from 1956 to 2020. The years below the cultivar names represent the years of name release. In this diagram, solid lines denote direct lineage connections between progeny and recipients, whereas dashed lines indicate indirect associations within the same pedigree structure. del, mutation allele with a >380‐bp deletion in the first exon of *GA20ox2*.

Our analysis of Japanese historical field records of elite temperate *japonica* cultivars revealed that multiple‐copy *GA20ox1* and functional *Hd1* both tended to promote higher yield with *ga20ox2* among modern Japanese cultivars. This raises the question of when and how favorable combinational genotypes (multi/del/non‐ins) of *GA20ox1*, *GA20ox2*, and *Hd1* were preserved and incorporated into Japanese breeding. To answer this, we examined the historical and geographical emergence of temperate *japonica* cultivars carrying the favorable combinational genotypes from early to modern Japanese breeding programs. Among 58 Japanese cultivars for which the breeding region and the year of name release were known, 20 showed the consistency with the presence of both the multiple‐copy *GA20ox1* variant and a functionally strong *Hd1* allele, combined with the deletion‐type *ga20ox2* allele (Figure S10a). The origin of the multi/del/non‐ins genotypes was traced back to the late 1980s (Figure S10b,c). Following the release of Kinuhikari (Tabuchi et al., 2000), which harbors the favorable combinational genotypes of *GA20ox1*, *GA20ox2*, and *Hd1* (Figures S11–S13), in the Chubu region in the 1980s (Figure S14), cultivars of the multi/del/non‐ins genotypes were bred widely across rice‐growing regions in Japan, particularly from the east‐central to southwestern regions from the 2000s onward (Figure 5; Figure S15, Tables S6 and S7). Notably, among the above 58 cultivars, none of the cultivars bred in Tohoku or Hokkaido carried the deletion‐type *ga20ox2* allele (Figure S14; Table S6).

## DISCUSSION

### Possible roles of favorable combinational genotypes of *GA20ox1* , *GA20ox2* , and *Hd1* in balancing shoot growth, shaping semi‐dwarf architecture, and achieving high yields in modern Japanese rice‐breeding programs

Dwarfing has been central to modern rice breeding for improved lodging resistance and yield, but excessive dwarfism can reduce vegetative biomass and limit yield (reviewed by Nagai et al., 2018; Weng et al., 2025). Through the characterization of identified QTLs, we found that multiple‐copy *GA20ox1* genotypes positively influenced canopy height during the vegetative stage and functionally strong *Hd1* genotypes positively influenced shoot biomass (Figure 2 and Figure S8). These functionally strong genotypes are well conserved in temperate *japonica* cultivars, in contrast to tropical *japonica* and *indica* subspecies (Figure 3), suggesting that they play important roles in the breeding of elite temperate *japonica* cultivars. As expected, cultivars harboring both functionally strong *GA20ox1* and *Hd1* genotypes, along with the non‐functional (deletion‐type) *ga20ox2* allele, showed no biomass penalty under Japanese field conditions compared with those with functional *GA20ox2*, and they maintained CLs within the 60‐ to 80‐cm range typical of modern elite Japanese cultivars (Figure 4, Shimono et al., 2002). In contrast, combinations of functionally weak *hd1* and *ga20ox2* alleles resulted in reduced shoot biomass, and this may explain their limited co‐conservation among elite Japanese cultivars (Figure S10). Notably, combinational genotypes involving single‐copy *GA20ox1* and *ga20ox2* were absent from our genomic dataset of 58 Japanese cultivars. These findings strongly suggest that multiple‐copy *GA20ox1* and functionally strong *Hd1* genotypes are key partners with dwarfism‐related variants, enabling the development of semi‐dwarf cultivars without compromising shoot growth.

We next discuss how yield is influenced by the combinational genotypes of *GA20ox1*, *GA20ox2*, and *Hd1*. Our analysis revealed that cultivars carrying multiple‐copy *GA20ox1* and a functional *Hd1* allele in combination with *ga20ox2* produced higher yields than those with *GA20ox2* (Figure 4; Figure S9). As reviewed by Weng et al. (2025), maintaining CH in short‐culm ideotypes is one of the promising strategies for improving rice yield potential. This is because dwarfing raises the harvest index by allocating carbon away from elongating stems toward reproductive sinks, and traits that sustain biomass (e.g., an adequately tall canopy) preserve the spikelet sink strength (Fu et al., 2011; Weng et al., 2025). To increase canopy/plant height, multiple‐copy *GA20ox1* and a functionally strong *Hd1* allele should be helpful (Figure 2D; Figure S8d), thereby enhancing source strength. This may be one of the reasons why these functionally strong genotypes with *ga20ox2* increase yield in a wide range of Japanese regions.

### Geographical distribution of favorable combinational genotypes for breeding semi‐dwarf and high‐yielding cultivars across Japanese rice‐growing regions

In our 1936–2020 dataset of cultivars bred in the Hokkaido and Tohoku regions (i.e., northern Japan), no entries harbored the deletion‐type *ga20ox2*/*sd1* allele that confers IR8‐type semi‐dwarfism and is present in AD (Figures S7 and S14). This finding indicates that the *sd1*/*ga20ox2* variant derived from IR8 may have been used rarely in these regions. In our dataset, cultivars carrying the multiple‐copy *GA20ox1* and deletion‐type *ga20ox2* combination tended to show higher yields and shorter CLs, and this pattern held regardless of latitude (Figure S9). These observations suggest that neither yield potential nor CL is the main constraint on adopting this favorable genotypic combination in northern breeding programs. One possible explanation is that non‐functional *ga20ox2* natural variants may delay leaf development and reduce biomass accumulation during the vegetative stage under cooler, shorter growing seasons (Shimono et al., 2002). Under such conditions, breeders may have been reluctant to use dwarfism‐inducing variants unless they were combined with strongly favorable partner genotypes. This context likely contributed to the low frequency of the deletion‐type *ga20ox2* allele in cultivars bred in Hokkaido and Tohoku (Figure S14). Akihikari, which traces its pedigree back to Reimei (Ashikari et al., 2002; Futsuhara et al., 1967; Tomita & Ishii, 2018), provides a concrete example: despite this relationship, Akihikari does not carry the non‐functional *ga20ox2* allele (Table S6). This pattern indicates that, even when the Reimei‐derived *ga20ox2*/*sd1* allele was available in northern breeding populations, lines retaining it were not favored, probably because it impaired vegetative growth under the prevailing cool conditions.

Under these constraints on *ga20ox2*/*sd1* use, alleles that enhance yield without strongly penalizing growth in cool temperate climates become particularly important. Multiple‐copy *GA20ox1* has also been shown to increase yield in many modern Chinese *japonica*/*geng* cultivars, particularly in Liaoning Province, which lies at a latitude similar to Tohoku and Hokkaido (Wang et al., 2023). This independent evidence supports the idea that the *GA20ox1* tandem repeat can enhance yield potential even in cooler temperate rice‐growing regions. Overall, our results suggest that the tandem repeat natural variant of *GA20ox1* is a useful molecular target for marker‐assisted selection (reviewed by Jena & Mackill, 2008). With the markers developed in this study (Figure S11; Table S2), breeders can combine this variant with *ga20ox2* to develop semi‐dwarf, high‐yielding cultivars that perform well even under cool conditions in temperate rice‐growing regions of Asia.

## MATERIALS AND METHODS

### Development and cultivation of JAM2 lines

JAM2 lines were developed as described previously (Taniguchi, Sakamoto, et al., 2025). Briefly, four Japanese cultivars—Akidawara (AD), Iwaidawara (ID), Tachiharuka (TH), and Toyomeki (TM)—were used as the parents of JAM2 on the basis of their genetic diversity and regional breeding histories across Japan (Figure 1A, Figure S1). We first crossed AD with TM and ID with TH to produce seeds of two types. Then, these hybrids were crossed to produce four‐way recombinants. By using the single‐seed descent method, JAM2 were developed. One hundred JAM2 lines (F5 and F6) in 2022 and 2023 were used in this study. Seeds soaked in water at 30°C for 2 days were sown in trays filled with soil and incubated at 30°C in the dark for 2 days. Seedlings were grown in a paddy field in Kannondai, Tsukuba, for a month, and then, 33 seedlings per line were transplanted (11 plants 18 cm apart × 3 rows 30 cm apart, no replicates) into a nearby paddy field and cultivated for 5 months. The date of sowing was 18 April in 2022 and 17 April in 2023. Fertilizer was applied as basal dressing at a rate of 56:176:56 kg/ha for N:PO_4_:K. The JAM2 lines were grown at different locations within the same field across the 2‐year trials (Figure S2).

### SNP detection and haplotype inference

Whole‐genome resequencing reads were quality‐filtered and trimmed using Trimmomatic v0.39, and the cleaned paired‐end reads were aligned to the rice reference genome IRGSP‐1.0 using BWA‐MEM with default parameters. SAM files were converted to BAM format, merged, sorted, and indexed using SAMtools. PCR duplicates were marked using Picard, followed by local realignment around indels using GATK v3.7. High‐quality biallelic SNPs were identified from the realigned BAM files and used for downstream analyses. Haplotype inference was conducted following the method previously established for the Japan‐MAGIC population (Ogawa et al., 2021; Ogawa, Nonoue, et al., 2018; Ogawa, Yamamoto, et al., 2018). Briefly, parental haplotypes were inferred based on concordance between SNP genotypes of the 100 MAGIC lines and those of the founder cultivars using the reconstruction program, which implements a haplotype prediction algorithm originally developed for an Arabidopsis MAGIC population (Kover et al., 2009). In the haplotype inference, the parameter P was set to five (−p 5). Adjacent genomic regions assigned to the same founder were merged to define haplotype blocks, with block boundaries determined by changes in inferred parental origin. These haplotype blocks were used as categorical markers in subsequent haplotype‐based association analyses.

Considering the relatively small population size of the JAM2, we evaluated the statistical power and potential risk of false negatives in the haplotype‐based GWAS using Monte Carlo–based power simulations. The results are presented in Supplementary documentation S1.

### Phylogenetic analysis

Genome‐wide SNP data for 230 rice varieties were obtained from TASUKE+ for the Rice Allele Collection available in RAP‐DB (the Rice Annotation Project Database) (https://rapdb.dna.naro.go.jp). From these, SNP data for 85 Japanese cultivars were extracted for further analysis. Pairwise genetic distances among accessions were calculated as p‐distance. The resulting distance matrix was used to construct a Neighbor‐Joining (NJ) tree using the Neighbor program implemented in PHYLIP (version 3.697) (Felsenstein, 1989) with default parameters. The phylogenetic tree was visualized and annotated using Interactive Tree Of Life (iTOL) (Letunic & Bork, 2024).

### Phenotypic measurement and quantification

Days to heading (DTH) was scored as the number of days from transplanting rice to the field to the appearance of the first panicle in more than half of the plants in each JAM2 line. CL and panicle length (PL) of the longest culm on each plant were measured with a ruler, and panicle number (PN) was counted from 10 days to a month after heading. To determine PW and stem and leaf weight (SLW), shoots of mature plants were air‐dried for 1–2 months in a drying room and then cut 3 cm below the panicle base to separate the parts; the AGW was calculated as PW + SLW. The averages of five plants in the middle lane, except for the plants at both edges, were used as the CL, PW, SLW, and AGW values of each JAM2 line. CH was quantified by using images captured by an unmanned aerial vehicle (UAV) in our previous study (Taniguchi, Sakamoto, et al., 2025) and used in this study. We analyzed historical field records collected by the National Agricultural and Food Research Organization (NARO) for yield performance evaluation from 2017 to 2021 for 48 Japanese cultivars evaluated at up to 69 field stations across the Japanese archipelago, including part of the data used in previous studies (Matsushita et al., 2024; Taniguchi, Hayashi, et al., 2025). CL, shoot biomass, and estimated grain yield (t/ha) were aggregated as means computed within “cultivar × prefecture × year” combinations and used for subsequent analyses.

### RNA extraction

One most expanded leaf per JAM2 line was collected at 6 weeks after transplanting (July 1, 2022 and June 29, 2023) from 9:00 to 11:00 am, immediately frozen in liquid N_2_, and stored at −80°C. Frozen samples were crushed by using a multi‐specimen cell‐disruption device (BMS‐A20TP, Bio Medical Science, Shinjuku, Japan), and total RNA from each sample was extracted immediately by using an RNeasy Plant Mini Kit (Qiagen, Inc., Venlo, The Netherlands) in accordance with the manufacturer's protocol.

### Library preparation and data processing for transcriptome analysis

RNA‐sequencing (RNA‐seq) libraries were prepared by using a NEBNext Ultra II Directional mRNA‐seq kit for Illumina (New England Biolabs, Inc., Ipswich, USA) in accordance with the manufacturer's protocols. The libraries were sequenced by using Illumina NovaSeq X Plus on a 25B flow cell with paired‐end 150‐bp and unique dual‐index reads. The reads were preprocessed in CLC Genomics Workbench version 25.0 (Qiagen). Reads were first trimmed with the following parameters: quality limit, 0.05; maximum number of ambiguities, 2. Next, raw reads were mapped to the genome assembly and gene sets of “Os‐Nipponbare‐Reference‐IRGSP‐1.0” with the following parameters: mismatch cost, 2; insertion–deletion cost, 3; length fraction, 0.8; similarity fraction, 0.8; and maximum number of hits per read, 10. Then, uniquely mapped exon reads were counted. Genes with at least one raw read count in all of the library samples for each comparison were analyzed. Log_2_(fold change) and adjusted *P‐*values (Wald test using the “DESeq” function) were calculated from raw read counts by using the R package DESeq2 (version 1.42.1) in R software (version 4.3.2). Differentially expressed genes compared with the control group were extracted on the basis of adjusted *P‐*values (*P*adj) < 0.05.

### Haplotype‐based GWAS and expression GWAS

Haplotype‐based GWAS used the haplotype information at each SNP and phenotype data in non‐parametric ANOVA (Kruskal–Wallis rank sum test) in the “kruskal.test” package of R software. *P*‐values obtained from the statistical analysis were used for the Manhattan plot. To identify QTLs for phenotypes, we selected SNPs with *P‐*values < 1.0 × 10^−3^. In the case of consecutive selected SNPs, the SNP with the lowest *P*‐value defined the QTL unless the distance between them was more than 2 Mb. For the expression GWAS, the haplotype information and transcriptome data of 100 JAM2 lines were used. *P*‐values for each transcript were obtained from the statistical analysis in the same way as for the GWAS and used for the Manhattan plot for target genes.

### Genomic DNA extraction, size selection, whole‐genome sequencing, and assembly to detect tandem repeat structures

Genomic DNA was extracted from each JAM2 founder by using a Nucleobond HMW DNA Kit (Takara Bio., Inc., Kusatsu, Japan) in accordance with the manufacturer's instructions. To enrich the extracts for high‐molecular weight DNA and reduce the number of short fragments, the extracts were subjected to size selection with a Short Read Eliminator Kit (Pacific Biosciences, Menlo Park, USA), which removes DNA fragments shorter than 10 kb to improve long‐read recovery efficiency. For sequencing, 2.5 μg of size‐selected genomic DNA was used to construct libraries with a Ligation Sequencing Kit LSK114 (Oxford Nanopore Technologies KK, Tokyo, Japan). The resulting libraries were sequenced on an R10.1 flow cell on PromethION 24 (Oxford Nanopore Technologies KK). The raw POD5 data were transformed into fastq with the dna_r10.4.1_e8.2_400bps_fast@v5.0.0 model in Dorado 0.9.0 (https://github.com/nanoporetech/dorado). To obtain better quality reads, we used the default parameters for cultivar AD, and we used the SeqKit2 (Shen et al., 2024) with the command “seqkit seq ‐m 3000 ‐Q 10” to filter a minimum length of 3000 bases, with an average quality score of at least 10 for cultivars ID, TH, TM. PECAT (https://github.com/lemene/PECAT) with the default parameters was used to obtain a draft genome assembly. The contigs were polished with the SeqKit tool with the command “‐m 1 000 000” to filter a minimum length of 1 000 000 bp. We used Os‐Nipponbare‐Reference‐IRGSP‐1.0 (*O. sativa* L. ssp. *japonica* ‘Nipponbare’) as a reference genome assembly.

### Genotyping based on SNPs, DNA short‐read depth, and PCR‐based assay

Among the QTLs detected, we determined the genotypes for each candidate gene (described later) related to agronomic traits on the basis of neighboring SNPs (Table S1) that constructed haplotype differences in the JAM2 lines: the SNP at M5483 (Chr01: 5273347) for *Gn1a*, M27769 (Chr03: 1299595) for *SOC1*, M31650 (Chr03: 36136637) for *GA20ox1*, M14158 (Chr01: 38380261) for *GA20ox2*, and M53254 (Chr06: 9333525) for *Hd1*. To confirm the mutations—insertion, deletion, and repeated genomic structures—we performed a PCR‐based assay by using established primers (Table S2) for *Gn1a*, *GA20ox1*, *GA20ox2*, and *Hd1*. The allele difference in *SOC1* was confirmed by using the TASUKE+ multiple genome browser in RAP‐DB. Next, we estimated the functional differences of each of the *GA20ox1*, *GA20ox2*, and *Hd1* genotypes. For *GA20ox1*, read‐depth–based copy number variation inference from short‐read resequencing (often using genome‐normalized depth) was conducted as a genotyping approach (Fuentes et al., 2019; Zhao et al., 2020). The multiple‐copy type was defined as a functionally strong genotype, either with a locus to whole‐genome depth ratio greater than 2 (based on short‐read sequencing) or with a repeated genomic structure of the *GA20ox1* locus confirmed by PCR assay. Otherwise, it was considered a single copy. For *GA20ox2*, the non‐functional alleles carried a >380‐bp (length) deletion in the first exon, which originated from the cultivar Dee‐geo‐woo‐gen (Ashikari et al., 2002; Chandler, 1968). For *Hd1*, the non‐functional alleles were characterized by a 36‐bp (length) insertion in the first exon. These deletion‐ and insertion‐type mutations in *GA20ox2* and *Hd1*, respectively, can be recognized either in the TASUKE+ browser by using short‐read depth information or by PCR‐based assay. Using 58 Japanese cultivars for which the year of name release and breeding region were recorded, we determined the combinational genotypes of *GA20ox1*, *GA20ox2*, and *Hd1* according to the criteria described above.

## AUTHOR CONTRIBUTIONS

HF: Writing—original draft and review & editing, data acquisition, data curation, data analysis, visualization, and conceptualization. AF: Data acquisition, data curation, and data analysis. TS: Data acquisition and data curation. YK: Data acquisition, data curation, and data analysis. KN: Data acquisition and data curation. TK: Data acquisition. JY: Writing—review & editing, data acquisition, data curation, data analysis, conceptualization, and supervision. DO: Writing—review & editing, data acquisition, data curation, data analysis, visualization, conceptualization, and supervision.

## CONFLICT OF INTEREST

No potential conflict of interest was reported by the author(s).

## Supporting information


**Figure S1.** JAPAN‐MAGIC2 founders. Founders of JAPAN‐MAGIC2 (JAM2). A rice plant of each cultivar was brought from the paddy field at the ripening stage and photographed. White bar, 50 cm.
**Figure S2.** Field experimental design in this study. 100 JAM2 lines and the four parental cultivars (AD, Akidawara; ID, Iwaidawara; TH, Tachiharuka; and TM, Toyomeki) were cultivated. The yellow frames indicate the location of the paddy field used in this study. The lists below the images show the actual order of rice plants. For each cultivar and line, a total of 33 plants were grown in a layout of 3 columns × 11 rows. Images were obtained on (a) August 22, 2022 and (b) August 22, 2023.
**Figure S3.** Distribution of single nucleotide polymorphism in each chromosome of JAM2 lines. (a) Proportions of founder‐derived single nucleotide polymorphisms (SNPs) across chromosomes 1–12 in JAM2 lines. Founders are categorized as Akidawara (AD), Iwaidawara (ID), Tachiharuka (TH), and Toyomeki (TM). (b) Frequency of SNPs along chromosomes 1–12. Frequencies are color coded: AD (black), ID (blue), TH (red), and TM (green).
**Figure S4.** Putative QTLs associated with agronomic traits conserved in JAM2 founders. (a) Distributed values of observed agronomic traits across JAM2 lines in 2022 and 2023 (*n* = 100). (b) Map of putative QTLs determined in this study. Each roman numeral represents a locus in Table 1. (c) Mean values of each trait calculated after classification by using the genomic regions of putative QTLs. These regions are shown in Table 1. PL, panicle length; CL, culm length; DTH, days to heading; SLW, stem and leaf dry weight; AGW, aboveground dry weight.
**Figure S5.** Gene structure of an insertion‐type *gn1a* variant conserved in Iwaidawara. (a) Scheme of novel natural variant of *Gn1a*. Black boxes indicate the exons, white boxes indicate untranslated regions, and black lines connecting black boxes indicate the introns. The inserted sequence of 535 bp and predicted amino‐acid sequence (blue letters) in the third exon of the Iwaidawara allele are shown. (b) Genome‐wide association study detecting the peak including the *Gn1a* locus in the JAM2 population in 2022 and 2023. Each plot represents a single nucleotide polymorphism. (c) Panicle length (PL) among JAM2 lines classified by the haplotype including the *Gn1a* locus. Means ± SD are shown (*n* = 16–33). Different letters indicate significant differences among JAM2 lines (*P* < 0.05, Tukey–Kramer test). AD, Akidawara; ID, Iwaidawara; TH, Tachiharuka; TM, Toyomeki.
**Figure S6.** Effect of the putative QTL corresponding to SOC1 on days to heading. (a) Visualization of *SOC1* variants among the four founders, Koshihikari (“Koshi”), and Aikoku. Polymorphisms and indels relative to Nipponbare are highlighted using different colors and symbols. (b) Genome‐wide association study (GWAS) for days to heading (DTH) detecting the peak including the *SOC1* locus among JAM lines. (c) DTH among JAM2 lines classified by haplotypes including the *SOC1* locus. Means ± SD are shown (*n* = 12–36). Different letters indicate significant differences among genotypes (*P* < 0.05, Tukey–Kramer test). (d) Expression GWAS of *SOC1* among JAM2 lines. (e) Differences in the mRNA levels of *SOC1* between lines harboring the Toyomeki‐derived *soc1* allele and those harboring the other founder‐derived *SOC1* alleles. Asterisks indicate significant differences between the haplotypes of Toyomeki and the other founders among the JAM2 lines (*P*adj < 0.05, *n* = 19–81). AD, Akidawara; ID, Iwaidawara; TH, Tachiharuka; TM, Toyomeki, FC, fold change.
**Figure S7.** Time series of canopy height at the vegetative stage in JAM2 lines classified by the haplotype including the *GA20ox2* locus. (a) Variation of *GA20ox2* genotypes derived from Akidawara (AD), Iwaidawara (ID), Tachiharuka (TH), and Toyomeki (TM). Time series from the middle of May to late August of (b) canopy height (CH, cm) and (c) days to heading (DTH) in JAM2 lines classified by the haplotype including the *GA20ox2* locus. Plants were transplanted to experimental fields on May 18, 2022 (0 DAT) and May 17, 2023 (0 DAT). Means ± SD for CH at two time points, and DTH (*n* = 23–26), are shown. Time series of CH are shown with means of each group classified by *GA20ox2* genotypes derived from each JAM2 founder.
**Figure S8.** Effects of the putative QTL containing *Hd1* on days to heading, culm length and shoot biomass. (a) Variation of *Hd1* genotypes derived from Akidawara (AD), Iwaidawara (ID), Tachiharuka (TH), Toyomeki (TM), Koshihikari (Koshi), and Kasalath (Kasa). (b) Days to heading (DTH), aboveground weight (AGW), and culm length (CL) among JAM2 lines classified by haplotypes including the *Hd1* locus. Means ± SD are shown (*n* = 20–28). Different letters indicate significant differences among cultivars (*P* < 0.05, Tukey–Kramer test). (c) mRNA levels of positive flowering regulators in JAM2 lines: *Early heading date 1* (*Ehd1*), *heading date 3a* (*Hd3a*), and *RICE FLOWERING LOCUS T 1* (*RFT1*). Means computed depending on the haplotype groups are shown (*n* = 20–28). Asterisks indicate significant differences between the haplotypes of ID and TM versus AD and TH among JAM2 lines (*P*adj < 0.05, *n* = 45–55) (d) Rice plants and (e) time series of AGW in Koshi and an *Hd1* near‐isogenic line (NIL) grown under experimental field conditions in 2023. The NIL harbors an insertion‐type mutation of the *hd1* allele in the Koshi genetic background. NIL plants headed at 58 days after transplanting (DAT) and the Koshi plants headed at 75 DAT. Means ± SD are shown (*n* = 5). Asterisks and numbers above each bar indicate significant differences (*P* < 0.05, Student's *t‐*test) and *P‐*values, respectively. Scale bars, 100 cm.
**Figure S9.** Culm length and yield affected by combinational genotypes of *GA20ox1*, *Hd1*, and *GA20ox2* in experimental fields from 2017 to 2021. (a) Culm length and (b) yield of 44 cultivars, classified by *GA20ox1*, *Hd1*, and *GA20ox2* combinational genotypes. The results in these cultivars are consistent with the analysis shown in Figure 4. Means in each region per year are shown in a combinational genotype‐dependent manner. multi, multiple copy; non‐del, non‐deletion; del, deletion‐type mutation; non‐ins, non‐insertion; ins, insertion‐type mutation.
**Figure S10.** Historical emergence of combinational genotypes of *GA20ox1* and *GA20ox2*, and of *Hd1* and *GA20ox2*. (a) Venn diagram of 58 Japanese cultivars classified according to *GA20ox1*–*GA20ox2* combinational genotypes or *Hd1*–*GA20ox2* combinations. multi, multiple copy; non‐del, non‐deletion; del, deletion. Historical emergence of combinational genotypes of (b) *GA20ox1* and (c) *Hd1* with *GA20ox2* among 58 elite temperate *japonica* cultivars bred in Japan.
**Figure S11.** PCR‐based assay for detecting duplicated region of *GA20ox1* locus. (a) Window of the TASUKE+ browser illustrating an estimated tandem repeat region including the genomic *GA20ox1* locus (Os03g0856700/LOC_Os03g63970) in the Japanese cultivars shown in Figure 5. The variation in read depth for each accession is represented by the black color gradient shown in the lower panel just below the center. An approximately 36‐kb genomic region from 36 127 kb to 36 163 kb shows higher read depth in cultivars harboring the estimated strong‐type *GA20ox1* genotype with a threshold of 2 × the average depth ratio (locus/the whole genome) and in Kinuhikari compared with Matsuribare (at the lowest position), suggesting the presence of a tandem repeat region in Kinuhikari. Pink highlighting from the upper to the lower panel represents the coding region of the Os03t0856700‐01 transcript. (b) PCR‐based assay to detect non‐repeat and tandem‐repeat genomic regions including the *GA20ox1* locus. Primer positions for distinguishing between different alleles are shown in orange.
**Figure S12.** PCR‐based assay to detect deletion‐type mutation of the *GA20ox2*/*Sd1* allele. PCR products are shown distinguishing *GA20ox2* (Os01g0883800/LOC_Os01g66100) alleles in the Japanese cultivars shown in Figure 5. Black boxes indicate the exons, white boxes indicate untranslated regions, and black lines connecting black boxes indicate the introns. Primer positions for distinguishing between different alleles are shown in orange.
**Figure S13.** PCR‐based assay to detect non‐functional *hd1* natural variants. PCR products are shown distinguishing *Hd1* (Os06g0275000/LOC_Os06g16370) alleles in the Japanese cultivars possessing the combinational genotypes of multiple‐copy *GA20ox1* and non‐functional *ga20ox2* (Figure 5). Black boxes indicate the exons, white boxes indicate untranslated regions, and black lines connecting black boxes indicate the introns. Iwaidawara and Toyomeki are control cultivars harboring *hd1* variants with a 36‐bp insertion in the first exon. Primer positions used to distinguish between different alleles are shown in orange.
**Figure S14.** Distribution of combinational genotypes of *GA20ox1* and *GA20ox2* in different Japanese regions at different ages. Fifty‐eight Japanese cultivars, classified by *GA20ox1* and *GA20ox2* combinational genotypes, are the same as those shown in Figure S10. multi, multiple copy; non‐del, non‐deletion; del, deletion.
**Figure S15.** Japanese breeding history of the development of rice cultivars harboring the multiple‐copy *GA20ox1* and functional *Hd1* with the IR8‐derived non‐functional *ga20ox2* combinational genotype. Geographical distribution of Japanese cultivars harboring the favorable combinational genotypes for high yield and shorter culm length across different Japanese regions from the 1980s to 2020. Favorable combinational genotypes are defined as a set of multiple‐copy *GA20ox1*, functionally strong *Hd1* alleles without a 36‐bp insertion in the first exon, and *ga20ox2* alleles with a >380‐bp deletion in the first exon. Each colored area denotes a region of Japan, ordered from north (Hokkaido) to south (Kyushu). Delineated areas within each region correspond to individual prefectures. Light green arrows indicate the distribution direction of favorable combinational genotypes from the east‐central to western regions of Japan, whereas orange arrows represent their spread toward the southwestern regions.

**Table S1.** Haplotype data for 100 JAM2 lines.
**Table S2.** Primers used in this study.
**Table S3.** Ratio of average depth between *GA20ox1* locus and the whole genome (*GA20ox1*/genome) in 220 varieties.
**Table S4.** *ga20ox2* natural variants determined by more than 380 bp deletion in the first exon in 220 varieties.
**Table S5.** *hd1* natural variants determined by a 36‐bp insertion in the first exon in 220 varieties.
**Table S6.** *GA20ox1* and *GA20ox2* combinational genotypes, year of name release, and breeding regions in 58 Japanese varieties.
**Table S7.** *Hd1* and *GA20ox2* combinational genotypes, year of name release, and breeding regions in 58 Japanese varieties.

**Data S1.** Supplementary documentation 1.

**Data S2.** Supplementary documentation 2.

## Data Availability

The datasets used and/or analyzed in the current study are available from the corresponding author.
