# Supplementary material for: Dissecting Agronomically favorable genotypes in temperate japonica rice via haplotype analysis of a Japan‐MAGIC population
Source: Plant J. 2026 Jun 4;126(5):e70936. doi: 10.1111/tpj.70936 (PMC13238393; doi:10.1111/tpj.70936)
Supplement: Supplementary file 3 — Data S1. Supplementary documentation 1. [file TPJ-126-0-s001.pdf]

Supplementary documentation 1

Statistical power analysis in Japan-MAGIC population

To evaluate the statistical power of the nonparametric tests used in our haplotype-based genome-wide association study (GWAS) and to assess the potential for statistical power and false negatives due to the relatively small population size, we conducted the Monte Carlo–based power simulations. Assuming a total sample size of 100 lines, corresponding to the size of the Japan-MAGIC 2 (JAM2) population derived from four founders, we simulated haplotype-group comparisons using the Wilcoxon rank-sum test (two groups) and the Kruskal–Wallis test (three or four groups). Haplotype-group sizes varied in steps of five individuals while maintaining the total sample size at 100. Simulated phenotypic data were generated from normal distributions with unit variance, assuming an adjacent group difference of one unit. For each haplotype-group configuration, 10,000 simulations were performed, and statistical power was estimated as the proportion of simulations in which the null hypothesis was rejected at a significance level of α = 0.05.

As a result, for two-group comparisons, statistical power was moderate (approximately 0.5 to 0.8) when the minimum group size was around 5 to 10 lines, but exceeded 0.8 once the minimum group size increased to approximately 15 to 20 lines and remained high thereafter (Supplementary documentation Fig. 1). In contrast, for three- and four-group comparisons, consistently high power (approximately 0.95 to 1.0) was observed across the tested range of minimum group sizes (approximately 5–50 lines). Under the present simulation setting, in which the adjacent difference between groups was fixed at one unit, increasing the number of groups resulted in greater overall separation among group distributions; thus, the observed power curves reflect both group configuration and the magnitude of between-group separation.

As illustrated in Fig. S3, the proportions of the four haplotypes were largely comparable and SNP loci with four haplotype groups were majority. Taken together, these results suggest that our haplotype-based GWAS achieves high statistical power with a limited risk of false negatives (Type II errors).

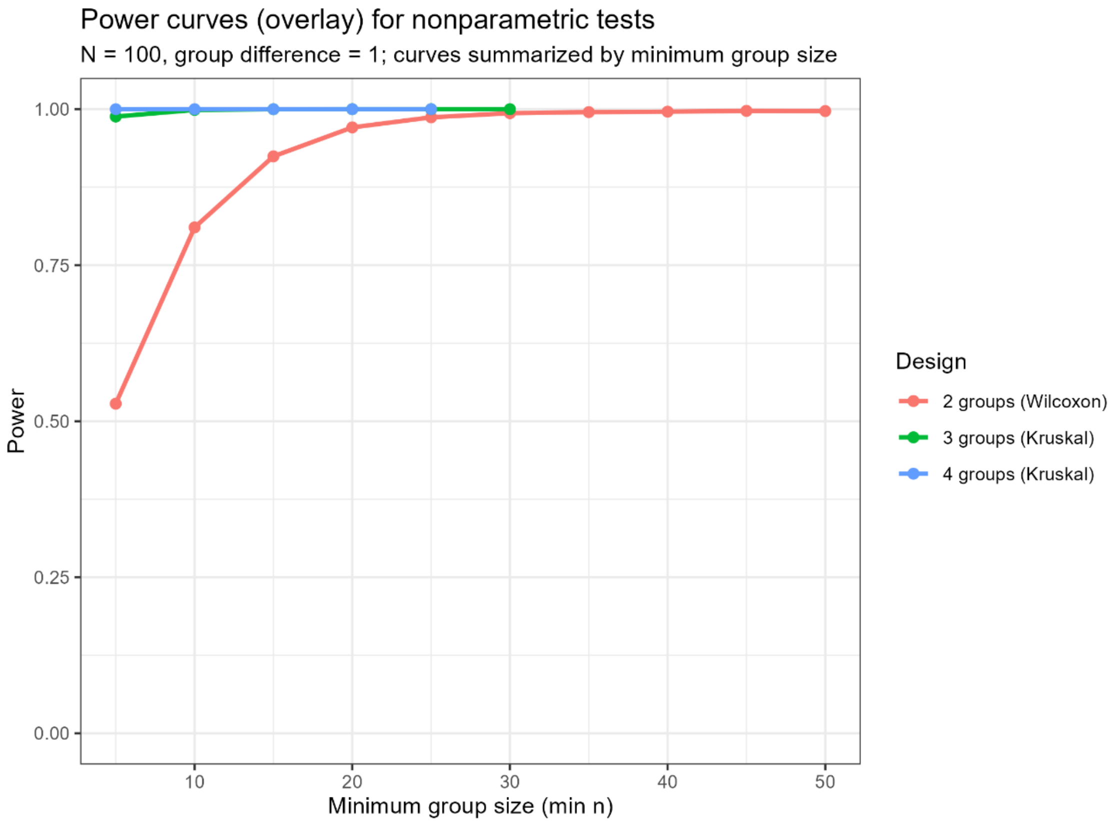

Supplementary documentation Fig. 1. Monte Carlo–based power analysis of nonparametric tests under the sample size used in this study. Power curves for the Wilcoxon rank-sum test (two groups) and the Kruskal–Wallis test (three and four groups) were estimated by Monte Carlo simulation assuming a total sample size of 100, corresponding to the size of the Japan-MAGIC 2 (JAM2) population analyzed in this study. Group sizes were varied in steps of five while maintaining the total sample size, and simulated phenotypic data were generated assuming an adjacent group difference of one unit. Statistical power was calculated as the proportion of simulations rejecting the null hypothesis at $\alpha = 0.05$. Power is plotted as a function of the minimum group size across groups to summarize multidimensional group-size configurations. All simulations and visualizations were performed using R software.
